# Analysis and Tools for Improved Management of Connectionless and Connection-Oriented BLE Devices Coexistence

**DOI:** 10.3390/s17040792

**Published:** 2017-04-07

**Authors:** Antonio Del Campo, Lorenzo Cintioni, Susanna Spinsante, Ennio Gambi

**Affiliations:** Dipartimento di Ingegneria dell’Informazione, Università Politecnica delle Marche, Via Brecce Bianche 12, Ancona 60131, Italy; S1080359@studenti.univpm.it (L.C.); s.spinsante@univpm.it (S.S.); e.gambi@univpm.it (E.G.)

**Keywords:** wireless sensor networks, BLE, discovery latency, connection-oriented network, connectionless network, home automation

## Abstract

With the introduction of low-power wireless technologies, like Bluetooth Low Energy (BLE), new applications are approaching the home automation, healthcare, fitness, automotive and consumer electronics markets. BLE devices are designed to maximize the battery life, i.e., to run for long time on a single coin-cell battery. In typical application scenarios of home automation and Ambient Assisted Living (AAL), the sensors that monitor relatively unpredictable and rare events should coexist with other sensors that continuously communicate health or environmental parameter measurements. The former usually work in connectionless mode, acting as advertisers, while the latter need a persistent connection, acting as slave nodes. The coexistence of connectionless and connection-oriented networks, that share the same central node, can be required to reduce the number of handling devices, thus keeping the network complexity low and limiting the packet’s traffic congestion. In this paper, the medium access management, operated by the central node, has been modeled, focusing on the scheduling procedure in both connectionless and connection-oriented communication. The models have been merged to provide a tool supporting the configuration design of BLE devices, during the network design phase that precedes the real implementation. The results highlight the suitability of the proposed tool: the ability to set the device parameters to allow us to keep a practical discovery latency for event-driven sensors and avoid undesired overlaps between scheduled scanning and connection phases due to bad management performed by the central node.

## 1. Introduction

Bluetooth Low Energy (BLE), marketed as Bluetooth Smart, is a subset of legacy Bluetooth (BT), sometimes referred to as classic Bluetooth (BT). It was introduced as a part of the Bluetooth 4.0 core specification [1]. While it inherits many features from the legacy BT, BLE is different in terms of all the functionalities regarding smart energy management. The BT radio is integrated into more than 8.2 billion products, produced by over 30,000 Bluetooth Special Interest Group (SIG) members [2], and this makes the BLE an ideal candidate for the wireless technology for Body Area Networks (BAN) and Internet of Things (IoT) markets, including automotive [3,4,5], smart cities [6], healthcare [7,8,9,10], fitness [11,12], consumer electronics [13], and building and home automation [14,15,16]. The advantages in the use of BLE include: (i) low power functionalities, operating for months or years on a coin-cell, (ii) small size and low cost, (iii) full compatibility with commonly-used devices such as mobile phones, tablets and computers. These properties make the BLE more favorable for many short-range communication applications. The classic Bluetooth has 79 channels, 1 MHz spaced, while the BLE splits the 2.4 GHz spectrum into 40 channels, each 2 MHz wide. Of these, 37 channels are used for data transmission, while the remaining *3* are used for connectionless communication, such as device discovery. In particular, the neighbour discovery has been designed according to a concise state-machine, aimed to simplify the operations and to minimize the power consumption. However, connectionless communication is also designed for generic broadcasting tasks, for example the transmission of *state data*, i.e., small and infrequent bits of data. This network architecture shall be referred to as the *connectionless data model*. It foresees the advertising devices, also called tags, which exchange tiny data with a scanning central device without synchronization, but at a reduced latency. It is the case for event-driven sensors, that need to send data only after event detection. Generally, these kinds of sensors are strongly energy-constrained and, for this reason, designed to keep the radio turned off for as long as possible. The BLE protocol recommends several parameter settings for connectionless and connection-oriented communications, and their proper tuning to balance and optimize the performance for a wide range of applications in terms of latency, energy consumption and throughput. Some application scenarios require that unconnected sensors that monitor relatively unpredictable and rare events, could operate in coexistence with sensors that are always connected, continuously communicating health or environmental data. The coexistence of connection-oriented and connectionless networks, sharing a single central/collector node can be required to reduce the number of handling devices, keeping the network complexity low and limiting the packet’s traffic congestion. The central device must be programmed in order to better schedule scanning and connection duty cycles, thus optimizing the use of the time division multiple access (TDMA) scheme. To the best of our knowledge, this is the first work that proposes a tool supporting the design and the configuration of BLE devices, allowing a single central node to manage either sensors working in connectionless mode, as advertisers, and other sensors, acting as slave nodes, in persistent connection. Models for both the connectionless and connection-oriented communications have been merged to obtain an algorithm that is able to estimate the discovery latency for event-driven sensors in relation to the loss of connected sensors data, caused by a bad central scheduling of communication operations. The paper is organized as follows: related works are presented in Section 2. An overview of BLE is provided in Section 3. In Section 4, first, a statistical-iterative model for discovery latency estimation is presented and, second, the tools to balance the performances of connectionless and connection-oriented devices, sharing a single central node, are provided. Section 5 presents the results obtained by the application of developed methods and provides an analysis of them. Finally, conclusions are given in Section 6.

## 2. Related Works

The related works section is divided into two parts: the former analyzes the literature focusing on the performance evaluation in device discovery, while the latter deals with connected devices issues.

### 2.1. Overview on Discovery Process Issues

Many works and several statistical and/or simulative approaches have been formalized to estimate the discovery latency in BLE networks.

In [17], the authors proposed an analytical model for a 3-channel-based neighbor discovery. Specifically, the model derives the discovery latency, not taking into account the collision amongst packets from homogeneous BLE devices as well as the interference with one or more channels. The model outcomes seem to match the simulation results for a scanning duty ratio greater than a certain value (0.3).

In [18] the authors propose an energy model for all the operating modes foreseen by the BLE protocol. Specifically, they provide a solution that is not closed-form, which estimates the mean discovery latency. The algorithm developed by the authors is at the basis of the model proposed herein and, for this reason some details will be provided below and a comparison among them discussed in Section 5.1.

In [19], an analytical model is developed to investigate the discovery probability. The authors have analyzed both the continuous and discontinuous scanning performed in active mode (i.e., with active exchange of scan request and scan response). According to the authors’ conclusion, the results, obtained via the proposed closed-form solution match the performed simulation experiments. The analysis of theoretical results appears to exhibit an exponential growth of device discovery delays, related to the increasing tags number. Despite this, the use of three advertising channels and tiny-sized frames has weak effects on the discovery latency.

In [20], starting from the model developed in [19], the authors present intensive simulations to investigate discovery probability and provide a quantitative examination of the influence of parameter settings on the discovery latency and the energy performance metric of the discovery process.

In [21], the authors propose a general model for analyzing the performance of neighbor discovery process in BLE networks. According to the Authors’ conclusion, the numerical results, produced by their model, meet the simulation outcomes for some parameter values specified by the standard.

### 2.2. Overview on Connection-Oriented Issues

In [22] the Author analyzes how the protocols BLE and Advanced and Adaptive Network Technology (ANT) may coexist on a single chip. Given that both ANT and BLE are low duty-cycle protocols, the cited work defines the general scheduling principles.

In [19] the authors investigate the impact of various critical parameters on the performances of BLE devices operating in connection mode. The paper provides experimental results that complement the theoretical and simulation findings, and indicates implementation constraints that may reduce the BLE performance.

In [8] the authors analyze a proof of concept of critical parameters setting in connection-oriented networks. The proposed system consists of two wearable BLE devices (smart shoes) that need to remain connected to a central node in order to stream data to it. The smartphone, often designated to be the central node in a *piconet* (i.e., connection-oriented network), is a commonly used device, that has sufficient capacity to jointly manage different radios (such as mobile radio, Bluetooth, WiFi, Near Field Communication (NFC), etc.) and/or a single radio serving sub-networks with different topologies and communication strategies. Starting from this key point, the following section will discuss how the central node can jointly manage BLE connected devices, and event-driven tags without a persisting connection.

## 3. Bluetooth Low Energy

**Figure 1:**
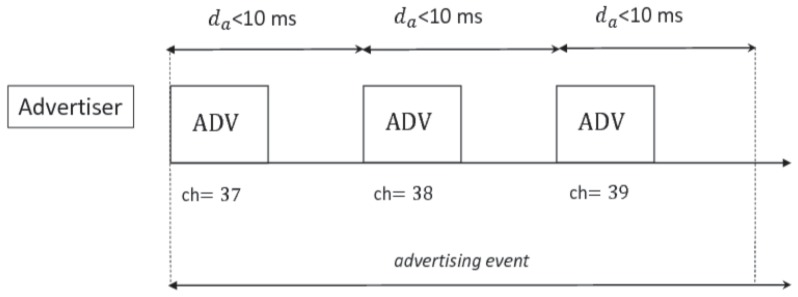
Timeline of an advertising event. It consists of three advertisements over channels 37, 38, and 39. After sending the advertisement message, the device remains on the channel for a random time up to 10 ms, listening to a connection request or a scan request for more data.

The BLE radio operates in the 2.4 GHz to 2.4835 GHz frequency Industrial, Scientific and Medical (ISM) band, with Gaussian Frequency-Shift Keying (GFSK) modulation. It splits the spectrum into 40 channels, each 2 MHz wide, and runs Frequency-Hopping Spread Spectrum (FHSS) to avoid interference. Three of these channels are used for advertising, connection-initiating and data-transfer in connectionless communication, while the remaining 37 channels are used for data-transfer in connection-oriented communication. In BLE, each advertising node runs an *advertising event* once at the beginning of each *advertising interval*. This can be set per tag in the range of values from 20 ms to 10,240 ms in steps of 0.625 ms. The BLE protocol does not implement carrier sensing on the used channels, and, because of this, the advertiser sends the same advertising packet to all the dedicated three channels 37 (2402 MHz), 38 (2426 MHz), 39 (2480 MHz) at each *advertising event* in order to avoid repeated collisions. Figure 1 depicts the *advertising event* timeline. Moreover, each advertiser adds a random delay up to 10 ms to avoid a massive contention.

**Figure 2:**
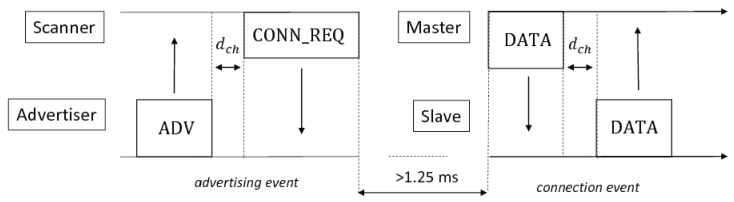
Timeline of connection procedure. After receiving the advertisement message, the central device, that intends to connect the advertiser, waits the inter-frame time 150 µs and, then, sends the connection request packet (CONN_REQ) on the same channel. After the CONN_REQ, the central, acting now as master, and the advertiser, acting now as slave, wait for a minimum of 1.25 ms before continuing at a data channel.

The advertising channels are between or outside the main frequencies used by IEEE 802.11 protocols, to prevent interference with the channels used by WiFi. In addition, the scanning devices run periodic operations, listening to advertisers during a *scan window*, once at the beginning of every *scan interval*. The *scan interval* and *scan window* can be set per scanner device. The scanner subsequently changes listening-channel at each *scan window*. The advertisers can operate in passive or active scanning. In passive mode, each node periodically broadcasts an advertisement message on the three channels. After sending, it can wait up to 10 ms before sending the same packet on the subsequent channel. The maximum payload per packet is 31 bytes long. The scanner does not reply to passive advertisers. On the contrary, in active mode, the scanner must reply immediately, unicasting the scan request message to the advertiser. The latter stops the messages exchange by broadcasting a scan response message (SCAN_RSP). Since the maximum payload of SCAN_RSP is 31 bytes, the advertiser doubles its transmission data capacity per *advertising event*. When a central device intends to initiate a connection with the advertiser, it sends a request (CONN_REQ) packet on the same channel used by the advertiser 150 µs after the reception of the advertisement message. The CONN_REQ payload contains the hopping map of channels that the connecting device must use in sequence during the connection. Thus, the advertiser immediately stops its *advertising event*, and jumps to the requested data channel to continue the connection sequence. Figure 2 depicts the connection procedure timeline.

Firstly, the two devices exchange information about the connection configuration. The master decides how often the slave has to wake up for incoming transmission (*connection interval* and *slave latency*). The choice depends on the slave and master availability and on the throughput requested by the particular application. After the initial configuration is completed, the devices can start the data transfer. In general, the slave can have multiple data transfers in a single *connection event*, with a maximum payload of 20 bytes.

## 4. Materials and Methods

Connectionless communication is mainly used to discover devices available for connection. As stated above, in some scenarios the advertisers might not require a connection, but can use the discovery procedure to exchange data with central devices without synchronization or acknowledgement mechanisms. The characterization of the time a sensor spends from the detection and transmission of an event to the successful delivery of a packet to the central node, boils down to the modeling of the discovery process. In the following discussion, the advertisers are indifferently referred to as tags. The main advantage of connectionless communication is the greater energy saving with respect to the connection-oriented communication, specifically in the case of few and unpredictable data to be exchanged. If an application requires that both the tags and the slaves share a single central node, proper parameters configuration is necessary to keep the *discovery latency* for tags low, while at the same time avoiding undesired overlaps between scanning and connection phases, due to a bad medium access management. Section 4.1 provides the description of the model for the discovery latency estimation; Section 4.2 formalizes the scheduling procedure the master implements to manage two slaves, and, finally, in Section 4.3 the connectionless and connection-oriented models are merged into an algorithm which aims to properly design the parameters configuration.

### 4.1. Connectionless Model

In connectionless networks, the devices are generally battery-powered; therefore, limiting the duration of transmission is necessary to save energy. An important metric to consider is the *discovery latency*, which is the time the tag spends in active state, from the start of transmission to the successful delivery of data to the central node. High *discovery latency* leads to increased energy consumption; therefore, limiting it is good practice. The proposed model for *discovery latency* estimation is based on [18], but it differs for the ability to reduce the algorithm computational complexity, and for an extensive stochastic approach to the problem description. As mentioned above, the tag periodically sends a burst of three packets on channels 37, 38 and 39. This operation is called *advertising event*. The interval between two *advertising events* is labeled as *advertising interval* and denoted by $T_{adv}$. Asynchronously, the scanner duty-cycles between scan and idle phases with period $T_{si}$, that is the *scan interval*. The scan duration per period is called *scan window* and denoted by $d_{sw}$. The scanner senses the 3 channels in a round-robin fashion. This implies that the duration of a complete scan is equal to $3T_{si}$. The *advertising interval* is the sum of two terms, as shown in Equation (1).

(1) $$T_{adv}\left(n\right)=T_{adv,0}+\rho \left(n\right)$$

$T_{adv,0}$ is a settable parameter, while ρ is a dynamic additive term, chosen randomly up to 10 ms each *n*-th *advertising event*. It is produced by a Random Number Generator (RNG) and has a double aim:

(i) reducing the probability of advertising packets collision,
(ii) preventing a tag from missing consecutive *scan events*.

**Figure 3:**
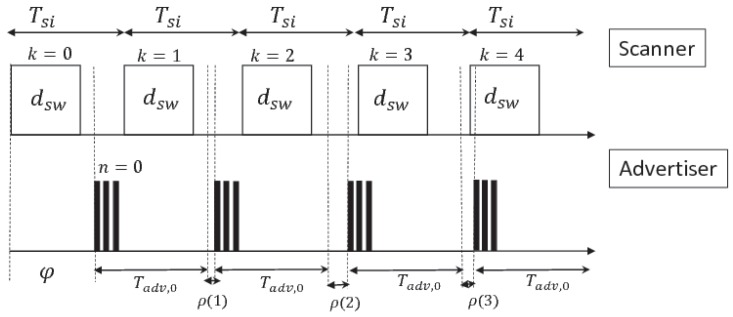
Timeline of the scanner-advertiser in the discovery procedure. The scanner duty-cycles transition from scanning phase to idle phase, listening to tag messages on the channel 37, 38 or 39. The tag (or advertiser) sends a burst of three packets on the three advertising channels in round-robin fashion. The advertising interval is composed of a static term $T_{adv,0}$ and a random additive term ρ(n).

Given the Equation (1) and the scanner-advertiser timeline in Figure 3, the beginning of *n*-th *advertising event* can be modeled by Equation (2).

(2) $$t_{a,n}\left(n,\phi \right)=\phi +n\cdot T_{adv,0}+\sum_{i=1}^{n}\rho \left(i\right)$$

where ϕ represents the offset between the beginning of the first *scan event* and the first *advertising event*. Setting out $T_{si}$ and $d_{sw}$ for the scanner and $T_{adv,0}$ for the tag, Algorithm 1 estimates the *expected discovery latency*, denoted by $d_{exp}$, by finding the couple of values (n,k) which verifies with high probability the condition expressed by Equation (3).

(3) $$k\cdot T_{si}-d_{early}\left(ch\right)\leq t_{a,n}\left(n,\phi \right)\leq k\cdot T_{si}+d_{sw}-d_{late}\left(ch\right)$$

where ch represents the listening channel of the *k*-th *scan window*, and is computed step by step as in Equation (4).

(4) ch(k)=mod(k,3)+37

The durations $d_{early}\left(ch\right)$ and $d_{late}\left(ch\right)$ represent the time before the beginning (the first) and the end (the second) of the *k*-th *scan event* that allows the *n*-th *advertising event* to fall within the *k*-th *scan window*; they are function of ch and, consequently, depend on *k* as shown in Table 1. It follows that the effective *scan window* $d_{sw}^{\prime }$ is shorter than $d_{sw}$. More in detail, its value is $\left(d_{sw}-d_{a}\right)$, where $d_{a}$ denotes the time for sending an advertising packet on a single channel and listening to a possible response. The protocol foresees that this time must be up to 10 ms, but usually, the value is much lower, almost 1 ms for passive scanning. In the case of active scanning this value can be larger, but always limited to 10 ms. At each *n*-th *advertising event* the tag randomly chooses ρ(n) through a pseudo random-number generator (RNG). In terms of probability the random variable ρ can be modeled through the uniform distribution with boundaries 0 and 10 ms. The *probability density function* (pdf) $f(t_{a,n})$ is computed as in Equation (2) taking into account that:

(i) ϕ, i.e., the offset between the first *scan event* and the first *advertising event* anchor points, is modeled through the uniform distribution with boundaries 0 and $\phi_{max}=3T_{si}$,
(ii) $\left(n\cdot T_{adv,0}\right)$ shifts $f(t_{a,n})$,
(iii) $\sum_{i=1}^{n}\rho \left(i\right)$ is the sum of *n* independent random variables ρ.

**Algorithm 1** Discovery latency estimation. The function takes as input arguments the scan interval $T_{si}$, the scan window $d_{sw}$ and the advertising interval $T_{adv,0}$, and gives back the expected discovery latency $d_{exp}$.
1:**function** discoveryLatency($T_{si},d_{sw},T_{adv,0}$)2:    $\rho_{max}$ ← 10 ms3:    $\phi_{max}$ ← $3T_{si}$4:    ϵ ← 0.00015:    *n* ← 06:    $p_{cM}$ ← 17:    $d_{exp}$ ← 08:    **while** $(p_{cM})\geq \epsilon$ **do**9:        $f(t_{a,n})$ ← $pdf(\phi +n\cdot T_{adv,0}+\sum_{1}^{n}\rho )$10:        $\mu_{f}$ ← $\frac{\phi_{max}}{2}+n\cdot T_{adv,0}+\frac{\rho_{max}}{2}$11:        $hw_{f}$ ← $getSigma(f\left(t_{a,n}\right))$12:        $k_{min}$ ← $floor(\frac{\mu_{f}-hw_{f}}{T_{si}})$13:        $k_{max}$ ← $ceil(\frac{\mu_{f}+hw_{f}}{T_{si}})$14:        $p_{hit}$ ← 015:        **for** $k=k_{min}$ to $k_{max}$ **do**16:              ch ← mod(k,3)+3717:              $\left(d_{early},d_{late}\right)$ ← getInterval(ch)18:              $d_{a,evt}$ ← getAdvEvntDuration(ch)19:              $t_{k,s}$ ← $k\cdot T_{si}-d_{early}$20:              $t_{k,e}$ ← $k\cdot T_{si}+d_{sw}-d_{late}$21:              $p_{k}$ ← $\int_{-\infty }^{t_{k,e}}f\left(t_{a,n}\right)-\int_{-\infty }^{t_{k,s}}f\left(t_{a,n}\right)$22:              $p_{hit}$ ← $p_{hit}+p_{k}$23:              $d_{exp}$ ← $d_{exp}+p_{k}\cdot p_{cM}\cdot (n\cdot \left(T_{adv,0}+\frac{\rho_{max}}{2}\right)+$ $d_{a,evt})$24:        **end for**25:        $p_{cM}$ ← $p_{cM}+\left(1-p_{hit}\right)$26:        *n* ← n+127:    **end while**28:    **return** $d_{exp}$29:**end function**


The pdf of the term (iii) is the convolution of *n* uniform distributions. For n=1 the pdf is a uniform distribution with boundaries 0 and 10 ms. For n=2 the pdf is a triangular distribution. In contrast, for n>2 the Central Limit Theorem (CLT) can be applied with sufficient approximation, describing the pdf of (iii) as a Gaussian distribution. The latter, shifted by (ii), takes $\mu_{g}=n\times 5$ ms as mean and $\sigma_{g}=\sqrt{\frac{n}{12}}\times 10$ ms as standard deviation. Finally, the pdf of $t_{a,n}$ isproduced by the convolution between the pdf of (i) + (ii) and the pdf of the random variable ϕ by (iii). Algorithm 1 for each *advertising event n* calculates the expected value $\mu_{f}$ and the half-width $hw_{f}$ of the distribution function $f(t_{a,n})$, that is the half time interval width, which at least 99% of $f(t_{a,n})$ falls within. These are used to evaluate $k_{min}$ and $k_{max}$, which are the lowest and highest indices of the *scan events*, that the *n*-th *advertising event* probabilistically overlaps with. The for-loop at each step, from $k_{min}$ to $k_{max}$, updates the channel ch, and consequently the time $d_{early}\left(ch\right)$, $d_{late}\left(ch\right)$ of the *k-th scan event* and the current duration of the advertising event, denoted by $d_{a,evt}\left(ch\right)$ and calculated as in Table 2. Then, the effective start and end time of the *k*-th *scan event*, $t_{k,s}$ and $t_{k,e}$ respectively are evaluated. Given those values, the algorithm calculates $p_{k}$, i.e., the probability for the *n*-th *advertising event* being received successfully by the scanner, according to Equation (7), as the difference between the cumulative functions Equations (5) and (6).

(5) $$F_{f(t_{a,n})}\left(t_{k,s}\right)=P\left(f(t_{a,n})\leq t_{k,s}\right)$$

(6) $$F_{f(t_{a,n})}\left(t_{k,e}\right)=P\left(f(t_{a,n})\leq t_{k,e}\right)$$

(7) $$p_{k}=F_{f(t_{a,n})}\left(t_{k,e}\right)-F_{f(t_{a,n})}\left(t_{k,s}\right)=\int_{-\infty }^{t_{k,e}}f\left(t_{a,n}\right)-\int_{-\infty }^{t_{k,s}}f\left(t_{a,n}\right)$$

For the calculation of $d_{exp}$, two other variables, $p_{hit}$ and $p_{cM}$, are required:

(i) $p_{hit}$ is the probability for a successful reception of the *n*-th *advertising event*, given that all previous events have not been received at the scanner,
(ii) $p_{cM}$ is the cumulative miss probability that *n* *advertising events* do not lead to a successful reception.

The joint probability $p_{cM}\cdot p_{k}$ gives the probability that the *n*-th *advertising event* is needed to close the cumulative miss probability $p_{cM}$ to zero. The probability $p_{cM}$ is the stop condition of the while-loop. In fact, when $p_{cM}$ reaches a value under the given threshold ϵ, the algorithm outputs the final estimation of $d_{exp}$. The estimation accuracy depends on ϵ: the smaller ϵ, the better the accuracy, but the higher the computational cost. The value 0.0001 for ϵ allows a good accuracy.

### 4.2. Connection-Oriented Model

The following discussion looks into the case in which the central node handles up to 2 connected devices. The *connection interval* is the interval time between two consecutive *connection events* and is denoted by $T_{ci}$. It must be set as multiple of 1250 µs. Within the connection phase, master and slave negotiate the connection parameters:

(i) *connection interval* ($T_{ci}$) from 7.5 ms to 4 s in steps of 1.25 ms,
(ii) *slave latency*, that is the number of connection events the slave could skip in lack of packets to send,
(iii) *supervision timeout*, maximum time the master must wait in the case of a lack of slave link before closing the connection.

**Figure 4:**
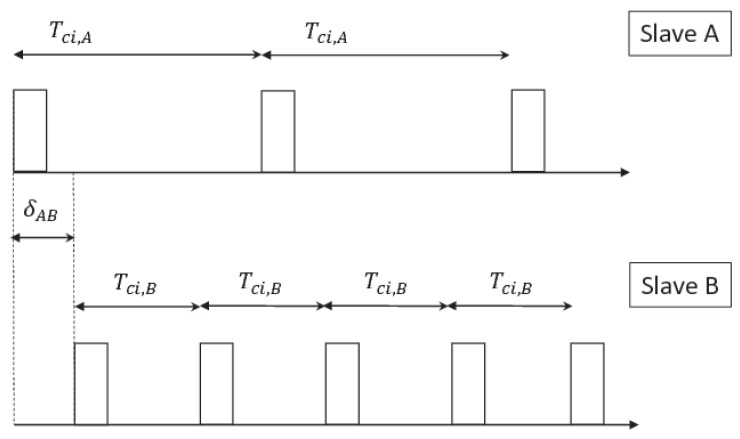
Master scheduling of slaves. The slaves A and B have connection intervals $T_{ci,A}$ and $T_{ci,B}$. In order to avoid connection events overlaps, the master must properly choose the initial time distance of B’s anchor point from A’s anchor point.

Given one device, denoted by A, connected to the master, if a second slave, denoted by B, requests to establish a connection with the same master, the latter must properly space between A’s and B’s anchor points in order to avoid the two connections experiencing colliding transmissions. When choosing the offset $\delta_{AB}$ (shown in Figure 4) the central node must take into account:

(i) the slave request, i.e., a minimum and a maximum value of acceptable $T_{ci}$,
(ii) the condition of *no-time-coincidence*, expressed by Equation (8).
  (8) $$gcd\left(T_{ci,A},T_{ci,B}\right)+\tau <\left(2\cdot gcd\left(T_{ci,A},T_{ci,B}\right)-\tau \right)$$

The first term in Equation (8) is the *greatest common divisor* (*gcd*) between the *connection interval* of A and B, τ is the sum of $d_{ce}$ and $d_{ch}$: the former is the duration of a connection event (up to 10 ms) and the latter is the channel hop duration.

Whether the condition Equation (8) is verified, the master has just to choose $\delta_{AB}$ within the interval shown in Equation (9), in order to avoid overlaps.

(9) $$m\cdot gcd\left(T_{ci,A},T_{ci,B}\right)+\tau <\delta_{AB}<\left(m+1\right)\cdot gcd\left(T_{ci,A},T_{ci,B}\right)$$

where *m* is an integer. If $\delta_{AB}$ does not fall within the interval given by Equation (9), overlaps will occur every $T_{ci,A(B)}/lcm\left(T_{ci,A},T_{ci,B}\right)$ transmissions of slave A(B). Else if the condition Equation (8) was not verified, any $\delta_{AB}$ the master would choose will result in collision. As a result, the master would not be able to handle two connections.

### 4.3. Coexistence Issues Modeling

The previous paragraph discussed the scheduling techniques the master must implement to avoid overlaps between two slaves transmissions. Here, we analyzed the coexistence of connectionless and connection-oriented devices interfacing a single central device. In this case, the central node should avoid not only the overlap of *connection events*, but also the coincidence between *connection* and *scan events*. In fact, if the central scheduled a *connection event* overlapping with a pre-scheduled *scan event*, the *connection event* would be skipped, with the resulting loss of the slave’s packet. It follows that an improper choice of scan parameters could result in an undesired loss of data from connected devices. The developed Algorithm 2 takes as input the *connection intervals $T_{ci,A}$ and $T_{ci,B}$*, the *advertising interval* $T_{adv,0}$, and the percentage of packets $p_{succ}$ the master must guarantee to exchange with the slaves without overlaps, and gives back the *scan interval* $T_{si}$, the *scan window* $d_{sw}$, and the derived *expected discovery latency* $d_{exp}$. Firstly, the algorithm checks the condition Equation (10). This is obtained from Equation (8) by adding the time necessary to switch from *connection event* to *scan event* and viceversa ($2\cdot d_{ch}$).

(10) $$gcd\left(T_{ci,A},T_{ci,B}\right)+\tau +2\cdot d_{ch}<2\cdot gcd\left(T_{ci,A},T_{ci,B}\right)-\left(\tau +2\cdot d_{ch}\right)$$

If this is not verified, the algorithm exits without results, otherwise it goes to the following steps. The offset $\delta_{AB}$ must be chosen as:

(11) $$\delta_{AB}=gcd\left(T_{ci,A},T_{ci,B}\right)+\tau$$

**Algorithm 2** Parameter designer tool. The function takes as input the connection intervals $T_{ci,A}$ and $T_{ci,B}$, the advertising interval $T_{adv,0}$, and the percentage of packets $p_{succ}$ the master must guarantee to exchange with the slaves without overlaps, and gives back the *scan interval*
$T_{si}$, the scan window $d_{sw}$, and the derived expected discovery latency $d_{exp}$.
1:**function** parameterDesigner($T_{ci,A},T_{ci,B},T_{adv,0},p_{succ},$)2:    $d_{ce}$ ← 10 ms3:    $d_{ch}$ ← 150μs4:    τ ← $d_{ce}+d_{ch}$5:    **if** $\left(gcd\left(T_{ci,A},T_{ci,B}\right)+\tau +2\cdot d_{ch}\right)<\left(2\cdot gcd\left(T_{ci,A},T_{ci,B}\right)-\left(tau+2\cdot d_{ch}\right)\right)$ **then**6:        $\delta_{AB}$ ← $gcd(T_{ci,A},T_{ci,B})+\tau$7:        *r* ← $lcm\left(T_{ci,A},T_{ci,B}\right)/min\left(T_{ci,A},T_{ci,B}\right)$8:        $T_{si}$ ← $min(T_{ci,A},T_{ci,B})$9:        $t_{start}$ ← $\delta_{AB}$10:        $t_{k,start}$ ← $max(T_{ci,A},T_{ci,B})$11:        **for** k=1 to *r* **do**12:              **if** $t_{k,start}\leq t_{start}$ **then**13:                  $t_{k,start}$ ← $\lceil t_{start}/max\left(T_{ci,A},T_{ci,B}\right)\rceil \cdot max(T_{ci,A},T_{ci,B})$14:              **end if**15:              **if** $\left(t_{k,start}-t_{start}\right)>min\left(T_{ci,A},T_{ci,B}\right)$ **then**16:                  $\vec{d_{sw}}\left(k\right)$ ← $T_{SI}-\left(\tau +d_{ch}\right)$;17:              **else**18:                  $\vec{d_{sw}}\left(k\right)$ ← $t_{k,start}-\left(t_{start}+\tau +d_{ch}\right)$;19:              **end if**20:              $t_{start}$ ← $t_{start}+T_{si}$21:        **end for**22:        $d_{sw}$ ← $SelectScanWindow(\vec{d_{sw}},p_{succ})$23:        $d_{exp}$ ← $DiscoveryLatency(T_{si},d_{sw},T_{adv,0})$24:        **return** $T_{si},d_{sw},d_{exp}$25:    **else**26:        **return** null27:    **end if**28:**end function**


Given that the A’s and B’s anchor points present a repetitive timeline pattern, for each $r=\;lcm\left(T_{ci,A},T_{ci,B}\right)/min\left(T_{ci,A},T_{ci,B}\right)$, the algorithm calculates $T_{si}$ as the minimum value between A’s and B’s *connection intervals*. Later, it evaluates the *r scan windows* $d_{sw}$, including them in the vector $\vec{d_{sw}}$. The function $SelectScanWindow(\vec{d_{sw}},p_{succ})$ finds the *scan window* duration that makes the master able to successfully receive at least the percentage $p_{succ}$ of packets exchanged with the slaves. Algorithm 2 can also be used fixing $T_{ci,A}$ and $T_{ci,B}$, varying the value of $T_{adv,0}$. The way the algorithm is used depends on which parameters can be adjusted and on network design requirements for the particular application scenario. Some results are highlighted in the Section 5.

## 5. Results

This section is divided into two paragraphs: the former provides some results on discovery latency issue, approaching the Algorithm 1, while the latter shows how to use Algorithm 2 and understand its results, with the aim to balance and optimize the networks setting.

### 5.1. Connectionless Model Results

**Figure 5:**
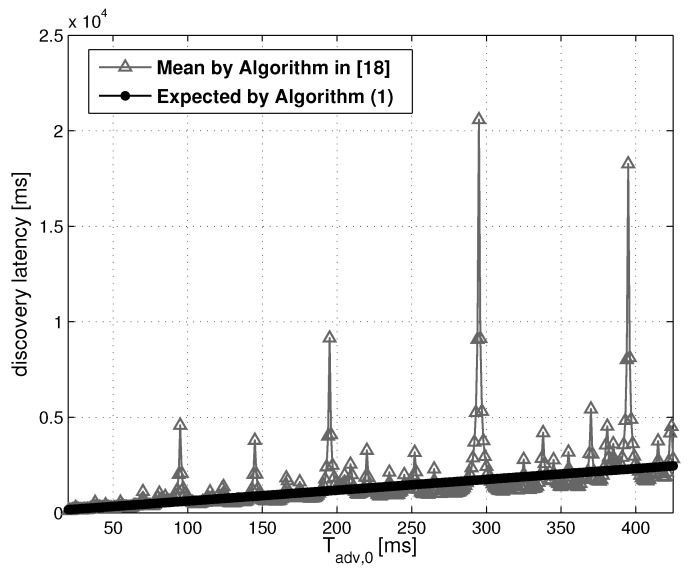
Comparison of models outcomes. The triangle-pointed line represents the mean discovery latency ${\bar{d}}_{mean}$ obtained by the model in [18]; the solid line represents the expected discovery latency $d_{exp}$ produced by Algorithm 1 for $T_{si}=100$ ms, $d_{sw}=25$ ms, $d_{a}=10$ ms.

Algorithm 1, proposed in this paper, estimates the discovery latency of a tag in advertising phase, not taking into account the collision amongst homogeneous BLE devices, the interference to one or more channels and the reception of corrupted packets. As widely explained in Section 4, it uses an iterative-probabilistic model which simplifies the one presented in [18]. In the following, we provide an example of a possible scenario, in order to discuss Algorithm 1 results, also comparing them to those of [18]. Table 3 summarizes the scanner and tag parameters designed if a tag self-advertises while one scanner is in listening mode. The Figure 5 compares the results obtained by the application of the model in [18] and Algorithm 1.

The solid line represents the expected discovery latency $d_{exp}$ produced by the former, while the triangle-pointed line represents the mean discovery latency, denoted by ${\bar{d}}_{mean}$, obtained by the model in [18]. The simulations are performed for the following configuration: $T_{si}=100$ ms, $d_{sw}=25$ ms, $d_{a}=10$ ms.

As visible in Figure 5, the amplitude of ${\bar{d}}_{mean}$ periodically becomes very high, giving rise to noticeable peaks for $T_{adv,0}$ close to multiples of $T_{si}$ and $d_{sw}$. It is important to clarify that the algorithm in [18] estimates the discovery latency averaging the delays obtained by varying the offset ϕ from 0 to $3T_{si}$. For $T_{adv,0}$ close to multiples of $T_{si}$ or $d_{sw}$, the discovery latency drastically increases, but only for a few values of ϕ. The average ${\bar{d}}_{mean}$ is strongly affected by these few but high values. Algorithm 1 generalizes the description of the discovery latency, not using any kind of average, but directly inserting ϕ in the probabilistic model. As a result, the solid line shows a linear relationship between $T_{adv,0}$ and $d_{exp}$ estimated by Algorithm 1. This result produces a manageable representation of $d_{exp}$ trend that is easier to integrate in more complex models.

### 5.2. Coexistence Model Results

When two devices request to establish a connection link to a central node, the latter must assign proper *connection intervals* to them and space their anchor points in order to manage the data exchanges without overlaps. In fact, assuming the master was previously connected to the slave A with *connection interval* $T_{ci,A}$, it must properly design the configuration for B for the incoming connection. The choice is constrained by the presence of one already active connection, and must fit the condition Equation (8). If the central node also has to listen to advertising devices, it must properly set the *connection interval* of the second device B and fit the condition Equation (10) in order to better schedule both the slaves and tags. Fixing $T_{ci,A}$ and $T_{adv,0}$, Algorithm 2 gives back the values of $T_{ci,B}$ that reach the right balance between a limited $d_{exp}$ and a high percentage $p_{succ}$ of successful *connection events*. The histogram in Equation (6) depicts the results produced from Algorithm 2, fixing $T_{ci,A}$ and $T_{adv,0}$ equal to the typically used value 100 ms and varying $T_{ci,B}$ in the interval [50÷600] ms with steps of 50 ms. It shows $T_{ci,B}$ and $d_{exp}$ on the *x*- and *y*- axis, respectively. There are three bars per each couple of values $\left(T_{ci,B},d_{exp}\right)$: black for $p_{succ}=100\%$, grey for $p_{succ}=80\%$, white for $p_{succ}=50\%$. Some observations can be derived:

(i) because of the condition of *no-time-coincidence* expressed by Equation (10) the master can accept only values of $T_{ci,B}$ multiple of $gcd(T_{ci,A},T_{ci,B})$; indeed, the histogram depicts bars only for values of $T_{ci,B}$ multiple of 50 ms,
(ii) the black bar is usually higher (or equal) than the grey one, and the latter greater (or equal) than the white one; this depends on the percentage $p_{succ}$ that must be guaranteed,
(iii) for some values of $T_{ci,B}$ the bars have similar height; in these cases the discovery latency $d_{exp}$ is not sensitive to $p_{succ}$, at least above a certain percentage threshold, e.g., for $T_{ci,B}=100$ ms the value of $d_{exp}$ is equal to 38.47 ms independently of $p_{succ}$ from 50% to 100%.

**Figure 6:**
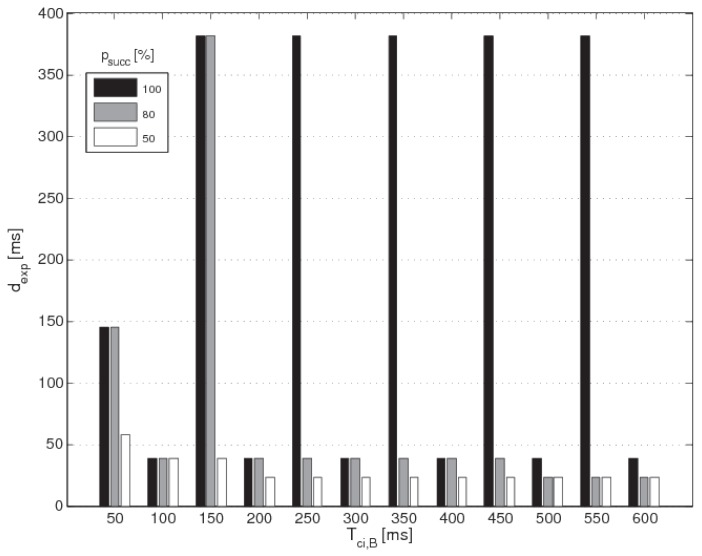
Results of the first parameters design. The histogram depicts $T_{ci,B}$ and $d_{exp}$ on the *x*- and *y*-axis, respectively. There are three bars per each couple of values $\left(T_{ci,B},d_{exp}\right)$: black for $p_{succ}=100\%$, grey for $p_{succ}=80\%$, white for $p_{succ}=50\%$.

The Table 4 gives the values of $T_{si}$ and $d_{sw}$ that produce the results shown in Figure 6. Notice that when $T_{ci,B}$ and $T_{ci,A}$ are multiples, the scanning duty cycle systematically overtakes the 50%, keeping $d_{exp}$ at low values. This fact points up that choosing the *connection intervals* equal or multiple to each other is a good practice to maximize the scanning duty cycle and, consequently, to limit the *discovery latency*.

**Figure 7:**
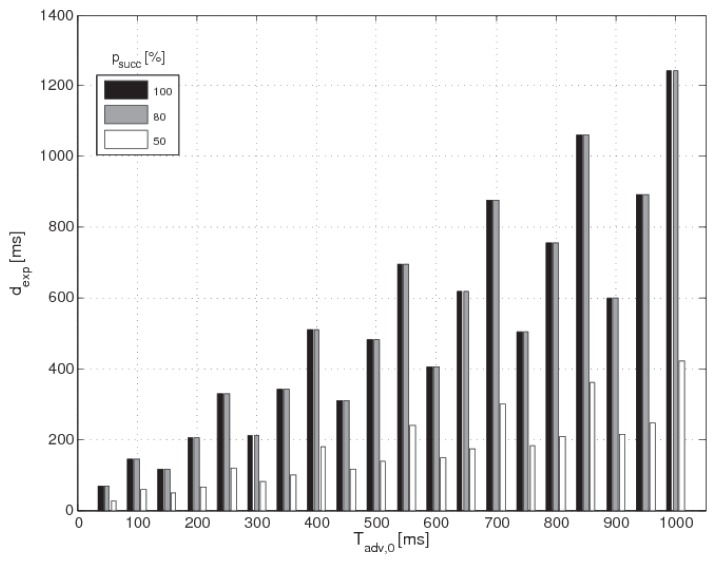
Results of the second parameters design. The histogram shows the *advertising interval* $T_{adv,0}$ on the *x*-axis and the *discovery latency* $d_{exp}$ on the *y*-axis. There are three bars per each couple of values $\left(T_{adv,0},d_{exp}\right)$: black for $p_{succ}=100\%$, grey for $p_{succ}=80\%$, white for $p_{succ}=50\%$.

Algorithm 2 can also be used by fixing $T_{ci,A}$ and $T_{ci,B}$ and varying $T_{adv,0}$. For example, assigning to the parameters $T_{ci,A}$ and $T_{ci,B}$ the values 100 ms and 50 ms respectively, the algorithm gives back the discovery latencies $d_{exp}$ related to ascending values of $T_{adv,0}$ in the range [20÷1000] ms, for given percentages of $p_{succ}$. For these settings the scan parameters are easily evaluated: $T_{si}=50$ ms, $d_{sw}=\;29.55$ ms for $p_{succ}\in \left\{100\%,80\%\right\}$ and $d_{sw}=39.70$ ms for $p_{succ}=50\%$. The histogram in Figure 7 shows the *advertising interval* $T_{adv,0}$ on the *x*-axis and the *discovery latency* $d_{exp}$ on the *y*-axis. There are three bars per each couple of values $\left(T_{adv,0},d_{exp}\right)$: black for $p_{succ}=100\%$, grey for $p_{succ}=80\%$, white for $p_{succ}=50\%$. As it can be seen, for fixed value of *scan interval* and *scan window* the *discovery latency* linearly rises by increasing the *advertising interval*, according to the results discussed in the previous paragraph. This implies that tags which transmit very frequently are able to minimize the *discovery latency*, particularly when the scan window is tiny compared to the *scan interval*, i.e., for a low receiver duty cycle.

### 5.3. Application in Home Automation and AAL Context

When using BLE in wireless Home Automation and AAL applications, the number of devices becomes a limitation as BLE implements a star topology and the central node can manage only a few devices in persistent connection. Due to the TDMA technique, the number of time slots available for connected devices is further limited id the application requires short connection intervals. The connectionless topology allows us to increase the number of sensors while reducing the connected nodes. On the other hand, the central node should run heavier scheduling operations to handle both the types of communications.

The presented tool is aimed at supporting the design and the configuration for these use cases. A possible scenario architecture is represented by the co-existence of the network systems presented in [8,23]. In the former, two wearable devices (smart shoes) are connected to a central node, while the latter shows a platform for assistive home technologies based on the Message Queuing Telemetry Transport (MQTT) protocol, which also foresees a gateway, acting as central node for BLE sensors. Different types of event-driven sensors installed in the house are in charge of detecting the events of interest, specifically:

- magnetic sensors placed on the entry door and on the windows, to detect opening and closing events;
- a Passive Infrared (PIR) sensor to be installed in the bathroom, to detect the user’s presence;
- a bed sensor placed under the mattress.

These sensors do not need persistent connection. For this reason, a connectionless communication is recommended. If the two cited networks require to share the central node, properly tuning some critical BLE parameters becomes necessary. As highligthed in [8], the smart shoes need to have the *connection intervals* equals to 100 ms and *slave latencies* equal to 7 *connection intervals*. As shown in Table 4, for $T_{ci,A}$ and $T_{ci,B}$ equal to 100 ms, the suggested values of scanning parameters, produced by Algorithm 2, are: $T_{si}=100$ ms and $d_{sw}=79.55$ ms. For these settings:

(i) the percentage $p_{succ}$ of connection events successfully run up to all those scheduled reaches the 100%;
(ii) the *expected discovery latency* will be 38.47 ms for sensors with *advertising interval* $T_{adv,0}$ equal to 100 ms.

The tiny value of the obtained *expected discovery latency*, quantitatively proves the suitability of this possible configuration and demonstrates that equal settings of slaves connection parameters maximize the time available for scanning operations. The increase in the number of the slaves makes $d_{sw}$ collapse. In an extended scenario, considering the example of more than a pair of smart shoes, that require persistent connection with an interval of 100 ms, the master could manage at most 4 pairs of devices at the same time, with the minimum offset $\delta_{AB}$ being equal to 10.150 ms, according to the condition Equation (11). In this case, the central node can use a maximum scan window of 17.650 ms to perform the discovery process. For this $d_{sw}$, Algorithm 1 estimates a value of discovery latency equal to 1015.4 ms for $T_{adv,0}=100$ ms, and 3860.4 ms for $T_{adv,0}=400$ ms. The former value of expected discovery latency implies the sensor is expected to self-advertise for almost 4 s, from the event detection to the successful delivery of data. This condition would cause a noticeable delay in the sensor feedback and an increased power consumption for the transmitting node.

With respect to slaves’ data reliability, the full compliance with the condition in Equation (11) and the adoption of the obtained *scan window* are enough to guarantee that $p_{succ}$ will reach 100%. Thus, in the case of slaves with equal *connection intervals*, the *connection events* will never overlap with *scan events*.

## 6. Conclusions

This paper proposed a tool aimed to support the design and the configuration of Bluetooth Low Energy devices, allowing a single central node to manage both the connected and the asynchronous sensors and/or actuators. The medium access management of the central node has been modeled, focusing on the scheduling procedure in both connectionless and connection-oriented communication. The developed models were then merged into a single tool. The results highlight the suitability of the proposed tool for a proper design of the device parameters, maintaining, for example, useful timing for discovery process of event-driven sensors and avoiding undesired overlaps between advertising events and connection events, due to inaccurate scanning and connection parameters configuration. The model of coexistence, proposed in this paper, considered only two devices in persistent connection. Future developments will be focused on the extension of the tool to a greater number of connected devices to verify how many different devices can be jointly supported by this kind of configuration.

## Figures and Tables

**Table 1:** Effective scan window parameters. The duration $d_{early}\left(ch\right)$ and $d_{late}\left(ch\right)$ are functions of ch and represent the time before the beginning (the first) and the end (the second) of the scan event, that allows the advertising event to fall within the scan window. $d_{sw}^{\prime }$ represents the effective scan window, which is shorter than $d_{sw}$.

Channel	$d_{\mathrm{early}}$	$d_{\mathrm{late}}$	$d_{\mathrm{sw}}^{\prime }$
37	0	$d_{a}$	$d_{sw}+d_{a}$
38	$d_{a}+d_{ch}$	$2d_{a}+d_{ch}$	$d_{sw}+d_{a}$
39	$2d_{a}+2d_{ch}$	$3d_{a}+2d_{ch}$	$d_{sw}+d_{a}$

**Table 2:** Current advertising event duration. It depends on ch and represents the current part of the *k*-th advertising event which the advertiser has already run.

Channel	$d_{a,evt}$
37	$d_{a}$
38	$2d_{a}+d_{ch}$
39	$3d_{a}+2d_{ch}$

**Table 3:** Scanning and Advertising configuration used for the example scenario.

Parameter	Value [ms]
$T_{si}$	100
$d_{sw}$	25
$T_{adv,0}$	[20÷425]

**Table 4:** Configuration for example scenario. The Table shows the values of $T_{si}$ and $d_{sw}$ produced by Algorithm 2 for $T_{ci,A}$ and $T_{adv,0}$ equal to 100 ms and varying $T_{ci,B}$ in the interval [50÷600] ms with steps of 50 ms.

Parameter	Value [ms]											
$T_{ci,B}$	50.00	100.00	150.00	200.00	250.00	300.00	350.00	400.00	450.00	500.00	550.00	600.00
$T_{si}$	50.00	100.00	100.00	100.00	100.00	100.00	100.00	100.00	100.00	100.00	100.00	100.00
$d_{sw},p_{succ}=100\%$	29.55	79.55	29.55	79.55	29.55	79.55	29.55	79.55	29.55	79.55	29.55	79.55
$d_{sw},p_{succ}=80\%$	29.55	79.55	29.55	79.55	79.55	79.55	79.55	79.55	79.55	89.70	89.70	89.70
$d_{sw},p_{succ}=50\%$	39.70	79.55	79.55	89.70	89.70	89.70	89.70	89.70	89.70	89.70	89.70	89.70

## Appendix A. Table of Symbols

**Models Parameters**		
**Symbol**	**Unit**	**Description**
$T_{adv,0}$	[s]	Static advertising interval, settable for the advertisers in the range from 20 ms to 10,240 ms in steps of 0.625 ms
ρ(n)	[s]	Dynamic additive delay up to 10 ms added to $T_{adv,0}$ for the *n*-th advertising event
$T_{adv}\left(n\right)$	[s]	Advertising interval value of the *n*-th advertising event, equal to $T_{adv,0}+\rho \left(n\right)$
ϕ	[s]	Offset between the beginning of the first scan event and the first advertising event
$T_{si}$	[s]	Interval between two consecutive scan events, settable for the scanners in the range from 0 to 10,240 ms
$d_{sw}$	[s]	Duration of active scanning for scan event, settable for the scanners in the range from 0 to $T_{si}$
ch	-	Advertising channel number: 37, 38, 39
$d_{early}\left(ch\right)$	[s]	Time before the beginning of the *k*-th scan event that allows the *n*-th advertising event to fall within the *k*-th scan window; depends on ch
$d_{late}\left(ch\right)$	[s]	Time before the end of the *k*-th scan event that allows the *n*-th advertising event to fall within the *k*-th scan window; depends on ch
$d_{a}$	[s]	Time for sending an advertising packet on a single channel and listening to a possible response, up to 10 ms
$d_{a,evt}$	[s]	Current portion of the *k*-th advertising event which the advertiser has already run.
$d_{sw}^{\prime }$	[s]	Effective scan window, equal to $\left(d_{sw}-d_{a}\right)$
$t_{a,n}\left(n,\phi \right)$	[s]	Anchor point of the *n*-th advertising event; also depends on ϕ
$f(t_{a,n})$	-	Probability distribution function of $t_{a,n}\left(n,\phi \right)$
$\phi_{max}$	[s]	Model boundary for ϕ equal to $3T_{si}$
$\mu_{X}$	-	Expected value of *X* probability density function
$\sigma_{X}$	-	Standard deviation of *X* probability density function
$hw_{X}$	-	Half time interval width, which at least the 99% of $f(t_{a,n})$ falls within
$k_{min}$	-	Lowest index of the scan events the *n*-th advertising event probabilistically overlaps with
$k_{max}$	-	Highest index of the scan events the *n*-th advertising event probabilistically overlaps with
$t_{k,s}$	[s]	Start time of the *k*-th scan event with duration $d_{sw}^{\prime }$
$t_{k,s}$	[s]	End time of the *k*-th scan event with duration $d_{sw}^{\prime }$
$F_{X}\left(X\right)$	-	Cumulative distribution function of a random variable X, representing the probability that the random variable X takes on a value less than or equal to X
$p_{k}$	-	Probability for an advertising event being received in the *k*-th scan event
$p_{hit}$	-	Probability for a successful reception of the *n*-th advertising event, given that all previous events have not been received at the scanner
ϵ		Model parameter representing the upper bound of $p_{cM}$
$p_{cM}$	-	Cumulative miss probability that *n* advertising events do not lead to a successful reception.
$d_{exp}$	[s]	Expected discovery latency for given $T_{si}$, $d_{sw}$ and $T_{adv,0}$
${\bar{d}}_{mean}$	[s]	Mean discovery latency calculated by the algorithm in [18]
$T_{ci}$	[s]	Interval between two consecutive connection events, settable in the range from 7.5 to 4000 ms in steps of 1.25 ms
$d_{ce}$	[s]	Duration of a connection event, up to 10 ms
$d_{ch}$	[s]	Duration of the time used for channel hopping
τ	[s]	Sum of $d_{ce}$ and $d_{ch}$
$\delta_{AB}$	[s]	Offset between A’s and B’s first anchor points
$P_{succ}$	[%]	Percentage of connection events successfully run up to all those scheduled
gcd(a,b)	-	Greatest common divisor of *a* and *b*
lcm(a,b)	-	Least common multiple of *a* and *b*
min(a,b)	-	Minimum value between *a* and *b*
max(a,b)	-	Maximum value between *a* and *b*

## Abbreviations

The following abbreviations are used in this manuscript:

AAL	Ambient Assisted Living
ANT	Advanced and Adaptive Network Technology
BAN	Body Area Network
BLE	Bluetooth Low Energy
BT	Classic Bluetooth
CLT	Central Limit Theorem
FHSS	Frequency Hopping Spread Spectrum
GFSK	Gaussian Frequency-shift keying
IEEE	Institute of Electrical and Electronics Engineers
IoT	Internet of Things
ISM	Industrial, Scientific and Medical
MQTT	Message Queuing Telemetry Transport
NFC	Near Field Communication
PIR	Passive Infra-Red
RNG	Random Number Generator
SIG	Special Interest Group
TDMA	Time Division Multiple Access
WiFi	Wireless Fidelity
